# Effect of fluoxetine on seizures in rats with high susceptibility to audiogenic stress

**Published:** 2021-04-22

**Authors:** Khadija Ismayilova, Ulduz Hashimova, Mohammad Reza Majidi, Farhad Rustamov

**Affiliations:** Academician Abdulla Garayev Institute of Physiology, Azerbaijan National Academy of Sciences, Baku, Azerbaijan

**Keywords:** seizure-tolerant and seizure-susceptible rats, tonic–clonic seizures, fluoxetine, serotonin, dopamine, noradrenaline, hypothalamus, frontal cortex

## Abstract

**Background:**

The seizures, triggered by a loud sound in laboratory animals, are associated with the imbalance of the brain monoamines. Serotonin (5-HT) is regarded as one of the principal neurotransmitters to be involved in regulation of wide variety of physiological and psychological processes. Among the drugs that affect 5-HT synaptic transmission, the leading role is given to selective serotonin reuptake inhibitors (SSRIs), such as fluoxetine.

**Aim:**

The aim of the study is to investigate the effects of fluoxetine on seizures in Wistar rats with high susceptibility to audiogenic stress.

**Methods:**

The study was performed on male Wistar rats. Before the experiments, the animals were tested for susceptibility to audiogenic seizures by exposure to a sound of 90–110 dB for 2 min in the soundproof box. The difference between congenital susceptibility to seizures served as a basis for dividing animals into two groups: Seizure-susceptible (SS) and seizure-tolerant (ST) rats. One hour before the experiment, the experimental animals were orally administered with fluoxetine, while the control rats were treated with distilled water. The effect of fluoxetine on the level of the biogenic amines in different regions of the brain was determined by enzyme-linked immunosorbent assay (ELISA).

**Results:**

It has been shown that in 100% of the cases, the control SS rats, exposed to audiogenic stimulus, exhibited wild running around in circles and jumping as some of the signs of seizure responses that evolved into tonic–clonic seizures in 70% of the cases. As regards the experimental SS animals: Only in 60% of the cases (P<0.05), they exhibited wild running and flinch, which did not evolve into the tonic–clonic phase.

**Conclusion:**

Decreased manifestation of seizures in the experimental SS rats under acute administration of fluoxetine may be explained by highly distinctive repertoire and spatial distribution of 5-HT receptors in their brain structures in comparison to the ST rats.

**Relevance for patients:**

The study of the monoaminergic mechanisms of seizure generation is of practical importance due to the existence of pharmacological agents, which selectively affect the activity of the monoaminergic systems of the brain, as well as the metabolism of the monoamines synthesized by them.

## 1. Introduction

The diagnosis and treatment of epilepsy in human are still major issues of neuropathology and psychiatry and understanding of the mechanisms of epileptogenesis is paramount to provide opportunities for new anti-seizure drug development. The cases of high resistance to anti-seizure therapy *(drug-resistant epilepsy)* in humans are often gene associated. In that context, it is of importance to study different aspects of epileptogenesis on animals with genetic predisposition to seizure *(seizure-susceptible animals)*. There are several strains of rodents including natural (Wag/Rij [Wistar Albino Glaxo from Rijswijk] – inbred strain) [1], synthetic (WAR [Wistar Audiogenic Rats] – outbred strain) [2], mutants and tottering rats (GAERS [Genetic Absence Epilepsy Rat from Strasbourg]) [3], which exhibit spontaneous seizures of different types (vs. chemically or electrically induced seizures). The seizures, triggered by a loud sound of specific modality in laboratory animals *(audiogenic epilepsy)*, are considered as one of the appropriate experimental models of human epilepsy [4]. This non-invasive model is interesting and reliable because of high reproducibility of original observations (obtained through applying this model). Such animals can be exposed to a sound more than once, that is, during the assessment of any effects on them, those animals can serve as their own control, which generally increases the reliability of the findings. Thus, some laboratory rat strains (KM and WAR) consistently exhibit seizures in response to auditory stimulus. However, the prevalence of seizure susceptibility to acoustic stimuli in other strains of rats, in particular Wistar rats, WAG/Rij is mere 15–20% [5].

It has been discovered that the brain monoaminergic systems are highly implicated in the pathogenesis of sound-induced seizures [6]. Much has been written on the abnormal neurotransmitter patterns underlying both depression and epilepsy. The large number of neurochemical studies has demonstrated the link between audiogenic epilepsy and the imbalance of monoamines [7]. Moreover, it has been shown that the nature of individual (phenotypical) reactivity of the central nervous system (CNS), exploratory and emotional behaviors, as well as a wide range of deviant behaviors was also associated with the peculiarities of the neurochemical organization of the brain, namely, the balance between brain monoaminergic systems [8]. Notably, Wistar rats, that are distinct in their individual susceptibility to acoustic stimulus, are normally characterized by different genetically determined ratio between brain noradrenergic, dopaminergic, and serotonergic systems activity. Seizure-susceptible (SS) rats were initially distinguished by the high level of dopamine (DA) and serotonin (5-HT), while seizure-tolerant (ST) ones had the high level of noradrenaline (NA) and the low level of 5-HT [8]. The level of the biogenic amines is determined to a large extent by the activity of enzymes involved in monoamine biosynthesis. The low level of NA and the high level of DA in the SS rats are assumed to be linked to the congenital deficiency of the dopamine beta-hydroxylase which is responsible for converting DA into NA, low activity of which correlates with audiogenic seizures [9].

The study of the monoaminergic mechanisms underlying seizure generation is of practical importance due to the availability of pharmacological agents which selectively affect the activity of the monoaminergic systems of the brain.

Serotonin is regarded as one of the principal neurotransmitters to be involved in regulation of wide variety of physiological and psychological processes, and changes in serotonin level and serotonin receptors activity profile can lead to psychoemotional disorders [10]. It is obvious that 5-HT deficiency is highly implicated in the generation of depression- and anxiety-related behavioral phenotypes through the impairment of serotonin synaptic transmission. Therefore, many of anxiolytics and antidepressants, commonly used in clinical practice, are targeted at the enhance of serotonin neurotransmission.

In this regard, of particular interest are the second-generation antidepressants – selective serotonin reuptake inhibitors (SSRIs), a class of drugs that are typically used in the treatment of major depressive disorder, anxiety disorders, and other psychological conditions. They are selectively aimed only at 5-HT and do not affect other chemical substances of the brain. The SSRIs primarily inhibit serotonin transporter (SERT), which is responsible for the reuptake of 5-HT into the presynaptic terminals. As a result, the concentration of 5-HT in the synaptic cleft increases as well as its availability for receptor binding, enhancing serotonin neurotransmission. Among SSRIs, the most widely prescribed antidepressant drug in the world is fluoxetine [11]. One hour after the administration of a single dose of fluoxetine, the level of extracellular 5-HT increases in many brain structures [12].

Considering the above, of particular interest is to study the effects of fluoxetine on seizures in Wistar rats with high susceptibility to audiogenic stress. The sound sensitivity indicator in our experiments was the severity and behavioral features of seizures in rats. Acoustically evoked seizure in rats is used as a model for elucidating the physiological and biochemical mechanisms of epilepsy and for looking of new ways of treatment and prevention of that disease [13].

However, it is known that epilepsy, as the system disease of the CNS, is accompanied by cognitive and behavioral impairment. Therefore, the clinical and experimental studies of epilepsy are mainly focused on comorbidity [14]. It was shown that the individual (and, apparently, genetic) variability plays a key role in comorbidity of anxiety and depressive disorders, which is confirmed by the clinical [15] and experimental [16] studies.

## 2. Methods

The study was performed on male Wistar rats (body mass of 250–300 g), which were bred at the Animal Research Facility of the Institute of Physiology, ANAS. Six to seven rats were housed per standard plastic cage (30 cm H × 40 cm W × 60 cm L) with room temperature at 22–24°C, and with food and water *ad libitum*. All experiments were performed in the morning between 10:00 a.m. and 1:00 p.m. All housing and testing were in accordance with the NIH guide for the care and use of laboratory animals (8^th^ edition, 2011). In addition, this research was considered and approved by the Scientific Council of the Institute of Physiology, ANAS on March 12, 2019 (Protocol No. 2) with the consent of the local ethics committee for the work with experimental animals.

Before the experiments, the rats were tested for susceptibility to audiogenic seizures. The animals were placed into the soundproof box and exposed to a sound of 90–110 dB. The duration of the sound signal was 2 min. In the case of clear development of epileptiform status, the sound was immediately switched off. Such limitation of the duration of acoustic stimulus prevents animals from death and development of large subdural hematoma [17].

The tolerance of animals for the effect of acoustic stimulus is different and reflects their innate ability to resist the effects of stressful stimuli. If the animals (they were the majority) did not exhibit any seizure-like behavior in response to a loud sound, except for the transient motor response (usually – flinching) or increased number of grooming episodes as indicators of emotional tension, then they were regarded as tolerant for the convulsiogenic sound effect. The rest of the animals, according to the developmental pattern of audiogenic seizures in rats [18], exhibited increase in motor activity (wild running and jumping) at first, after the short latent period. This stage of the seizures is called clonic running, followed by the clonic seizure phase (rhythmic intense muscle spasms accompanied by falling on abdomen). In our studies, the first and second stages of seizures were reported as generalized clonic seizures. Only the rats exhibiting the same responses 3–4 times in a row were selected for the experiments. Their difference in innate susceptibility to seizures served as a basis for dividing animals into two groups: Seizure-susceptible (SS) and seizure-tolerant (ST) rats. The registration of the stages of audiogenic seizures was used to select the groups of the same type from the general population of rats. Audiogenic seizures can be found in about 15–20% of Wistar rats [13].

For the study, 29 ST and 27 SS rats were used. Both types were subdivided into the experimental and control animals. One hour before the experiment, the experimental animals (ST (n=15), SS (n=14)) were orally administered through oral gavage with fluoxetine (Pharmascience, Montreal, Canada) at a dose of 25 mg/kg [19-21]. When taken orally, fluoxetine is absorbed well in the gastrointestinal tract (up to 95% of the taken dose). Fluoxetine is accumulated well in tissues and able to easily penetrate the blood–brain barrier. The control rats (ST (n=14), SS (n=13)) were treated with the equal volume of distilled water (diluent). During 2 days before the main experiments, the animals were handled for 5 min per day for avoiding stress response of rats to handling.

The effect of fluoxetine on the level of the biogenic amines in different regions of the brain (hypothalamus and frontal cortex) was determined by enzyme-linked immunosorbent assay (ELISA).

The experimental data were assessed for normality of distribution using the Kolmogorov–Smirnov test. In the case of normal data distribution, we applied the parametric Student’s t-test. If the data had large standard deviation, then the Mann–Whitney U-test was used. The statistical analysis was performed in a software package Statistica for Windows.

## 3. Results

**Figure 1:**
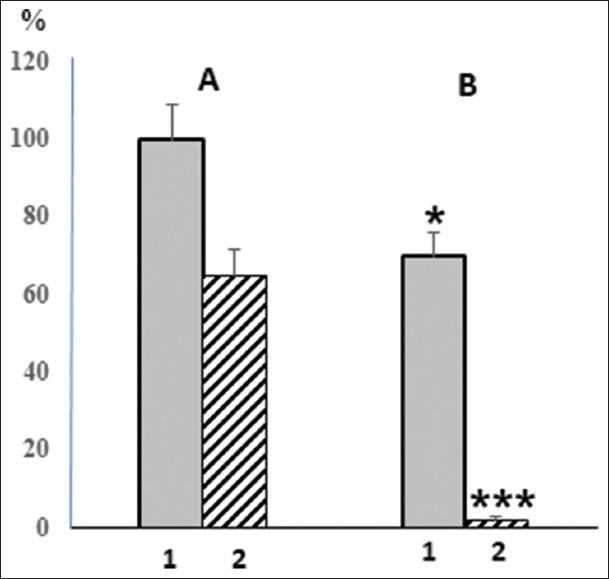
Manifestation (%) of the seizure stages in the SS rats under the effects of fluoxetine. A – running around in circles + jumping, B – tonic–clonic seizures; 1 – control animals, 2 – experimental animals. The reliability of the differences between the experimental and control animals was evaluated using Student’s t-test: *p <0.001.

**Figure 2:**
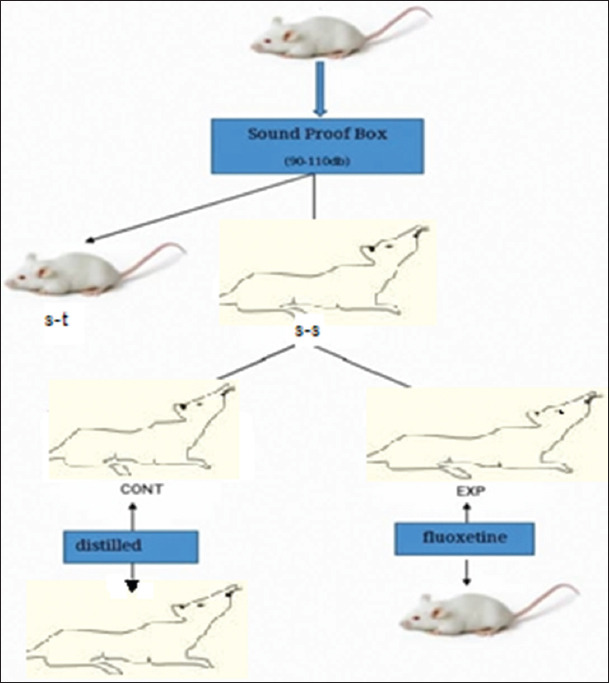
The effect of fluoxetine on seizure manifestation in rats with high sensitivity to audiogenic stress exposure.

This paper explores the effect of fluoxetine on manifestation of seizures in the rats susceptible to audiogenic stress. All the rats were tested for whether they exhibited both stages of seizures, as well as the motor seizure latent period. It has been shown that in 100% of the cases, the control SS animals, exposed to audiogenic stimulus, exhibited wild running around in circles and jumping as some of the signs of seizure responses that were evolving into tonic–clonic seizures in 70% of the cases (Figure 1). One hour after the single-dose administration of fluoxetine, the pattern of seizures changed dramatically. Thus, the severity of seizures decreased in the experimental animals: Only in 60% of the cases (*P*<0.05), they exhibited wild running and first flinch, which did not evolve into the tonic–clonic phase (Figure 2). Moreover, the effects of fluoxetine were clearly expressed in significant increase in the first flinch and motor seizure latent period in comparison with the control group of animals (93.8±12.3 and 49.6±9.2 s, respectively) (*P*<0.5), which showed all signs of seizure.

**Figure 3:**
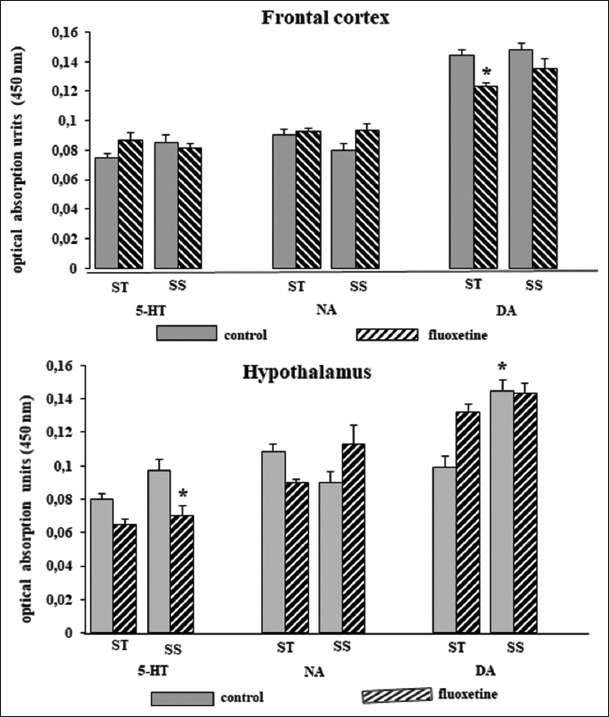
The levels of the monoamines in brain structures in rats with different phenotypes of the nervous system after administration of fluoxetine. The reliability of the differences between the experimental and control animals: *P*<0.05.

The study of monoamines in the hypothalamus and the frontal cortex of the brain revealed specific responses of the brain monoaminergic systems to acute administration of fluoxetine (Figure 3).

The outcome of fluoxetine administration depended on the individual properties of the CNS and the effects were distinct in different brain regions. It is known that the response of organism to stress is mainly determined by innate typological peculiarities of the higher nervous activity (animal behavior). There are the peculiarities of metabolism of neurotransmitters during chronic stress in the brain structures, the interaction of which determines the type of the nervous system of animal. These structures are hypothalamus and frontal cortex [22]. The mentioned structures play a key role in the formation of both voluntary and purposeful (emotional and motivated) behaviors. The hypothalamus and frontal cortex are involved in the regulation of animal behavior through the alteration of activities of the noradrenergic, dopaminergic, and serotonergic systems of the brain [23].

Thus, administration of fluoxetine-induced significant decrease in the level of 5-HT and reciprocal increase in the level of NA in the hypothalamus of the SS rats compared to the control animals. In the hypothalamus of the ST rats, there was decrease in 5-HT, accompanied by significant increase in DA. The decrease in the hypothalamic level of serotonin after the fluoxetine administration at a dose of 25 mg/kg may be explained by the fact that the latter reduces the expression of gene encoding tryptophan hydroxylase-2, which is responsible for the synthesis of 5-HT in the brain [20]. In the frontal cortex of the ST rats, however, administration of fluoxetine induced the tendency for 5-HT to increase, which was accompanied by significant decrease in DA that is involved in the control of anxiety [24] and regulation of motor activity as well [25].

Our findings are consistent with the works [26] demonstrating increase in 5-HT in the frontal cortex after administration of fluoxetine in the dose range of 3–154 mg/kg and with the fact that this increase can have a depressing effect on the dopaminergic system [27]. Perhaps, the main action mechanism of fluoxetine, according to some researchers, is based on the fact that this drug inhibits the reabsorption (reuptake) of extracellular 5-HT into the presynaptic neuron [28].

## 4. Discussion

The primary mechanism of action of fluoxetine is based on the fact that the drug binds to the serotonin transporter (SERT), thereby inhibiting the reuptake of extracellular 5-HT into the presynaptic terminal [28]. This leads to the increase in the level of 5-HT in the synaptic cleft and, as a result, to the enhancement and prolongation of its effects on the postsynaptic 5-HT receptors. A number of works showed that SERT blockade by fluoxetine resulted in significant decrease in the level of the primary metabolite of serotonin – 5-HIAA (5-hydroxyindoleacetic acid) in the brain [29]. The property of fluoxetine to increase the level of serotonin at the synapse allows this drug to be an effective treatment of depression and panic disorder. The drug selectively affects one of the serotonin receptors subtypes – 5-HT_1_, activity of which decreases with long-term use. As a result, fluoxetine, enhancing serotonergic transmission through the negative feedback mechanism, inhibits the 5-HT metabolism. Consequently, constant release of serotonin leads to the depletion of the serotoninergic system, which results in psychoemotional disorders. Positive therapeutic effect requires long-term use of fluoxetine – for weeks or even months. The delay in the manifestation of the therapeutic effect of fluoxetine is due to the time required for activation of the synthesis of 5-HT and the key neuronal enzyme – tryptophan hydroxylase 2. The inhibitor selectively targets only 5-HT and does not affect other brain chemicals.

Dose-dependent anticonvulsant effects of fluoxetine have been observed in genetically epilepsy-prone rats (GEPRs) and other animal models [30]. Thus, increased serotonin release through the administration of fluoxetine and 5-hydroxytryptophan to GEPRs enhances the anticonvulsant effect on them. The depletion of serotonin significantly reduces the anticonvulsant effect of fluoxetine. Moreover, it was reported that 6-methoxy-1,2,3,4-tetrahydro-beta-carboline (6-MeO-THBC) attenuates audiogenic seizures in 21-day-old DBA/2J mice, as well as inhibits brain monoamine oxidase (MOA) [31]. At the same time, the combination of the drugs, action of which corresponds to the action of 6-MeO-THBC, in particular a selective serotonin reuptake inhibitor (fluoxetine), with an MAO-A inhibitor suppressed audiogenic seizures more effectively than separately. However, it was shown that fluoxetine administered intraperitoneally, 30 min before pilocarpine, at two doses (10 and 20 mg/kg) had proconvulsant effects on rats with pilocarpine-induced seizures and status epilepticus [32]. Moreover, the findings of Aygun [33] demonstrate that the high dose of fluoxetine (intraperitoneally, 20 mg/kg) also significantly increased frequency and amplitude of epileptiform activity induced by penicillin. However, according to the contraindications of fluoxetine, its simultaneous use with other drugs has a depressing effect on the CNS, which, possibly, increases a risk of seizure.

On the other hand, in the work of the other researchers, acute administration of fluoxetine provoked no change neither in seizure susceptibility nor the glutamate release [34]. At the same time, it was found that rats susceptible to audiogenic seizures have congenital deficiency of GABAergic inhibition [35]. Thus, the inhibition process of generalized seizures in such rats after acute administration of fluoxetine is due to the activation of the serotonergic system of the brain, which has an anticonvulsant effect. In particular, acute administration of fluoxetine to the SS rats significantly decreases seizures. According to some experimental data, increase in the level of 5-HT, induced by fluoxetine, led to decrease in the frequency of seizures and reduction of the symptoms of depression [36]. Moreover, the clinical trials of the SSRIs showed that the patients with depression who received these drugs had few seizures than those who received placebo [37]. Perhaps, it is due to the fact that seizure is one of the symptoms of psychological disorders treated with antidepressants. In addition, according to another data, the effects of chronic fluoxetine treatment on rats of four genotypes pairwise different by susceptibility to audiogenic seizures and by anxiety or depression-like behavior indicate the key role of genetic background in the comorbidity of anxiety and depression with audiogenic seizures propensity [14].

In our previous experiments, the biochemical analysis of monoamines baseline level in the brain of rats with different stress sensitivity showed that normal animal behavior is maintained by proper balance between the serotonergic and catecholaminergic systems, and their activity imbalance is implicated in some characteristics of innate behavior [8]. The correlation analysis of the seizure severity and the level of the biogenic amines in the brains of the SS rats indicated that they had a deficit of NA, accompanied by increased metabolic rate of 5-HT in contrast to the animals that were tolerant to an acoustic stimulus. There is evidence that epileptiform seizures in animals may be caused by deficiency of the central noradrenergic transmission [38]. Hence, the baseline level of the biogenic amines in the brain structures behind the specific genetic and functional organization of the CNS [39] determines the nature of the stress response [40]. Hence, it was shown that epileptic seizures can be caused by deficiency of 5-HT in the brain [5]. As follows from our experiments, the baseline level of 5-HT in the brains of the SS rats is not high enough to prevent the development of seizures, and therefore, fluoxetine-induced raise of serotonin reduces seizure activity. In our view, high level of 5-HT in the brains of the SS rats is compensatory in nature and facilitates reducing of seizure activity in the SS rats, which do not apparently have genes determining tolerance to stress stimuli. The existence of such genes determining stress tolerance is indirectly indicated in the findings of Ungar who identified the low-molecular peptide in the brains of the rats – amelitin enhancing tolerance of the animals *(reducing of seizure activity)* to the effects of stress-related audiogenic signals [41].

The ST rats with low baseline level of 5-HT in the brain do not develop epilepsy, perhaps, due to the fact that non-prone to audiogenic seizures rats compared to prone ones have enhanced neuronal genetic activity containing high level of nuclear RNA [42].

Pronounced anti-seizure effect of fluoxetine, according to current scientific view, indicates that the activation 5-HT*_1A_* receptor induces a membrane hyperpolarization response caused by enhanced K^+^ conduction and thereby exerting anti-seizure effect on freely moving rats [43]. The anti-panic effect of fluoxetine is considered to be based on its capacity to selectively block 5-HT reuptake into presynaptic membrane, which leads to increase in the level of the neurotransmitter in the synaptic cleft and enhancement in the serotonin activity responsible for the development of anti-seizure effect. These findings are consistent with data, reported by another researcher, indicating an anti-seizure effect of fluoxetine and monoamine oxidase inhibitors (MAOIs) in genetically epilepsy-prone mice [44], thus supporting the idea that fluoxetine has a moderate stimulant effect in reducing worrying, anxiety, and a feeling of fear. Moreover, the study of the role of 5-HT in modulation of audiogenic seizures in genetically epilepsy-prone rats showed that the administration of a neurotoxin 5,7-dihydroxytryptamine (5,7-DHT) induced decreases in the frequency of audiogenic seizures in the animals due to the depletion of 5-HT [45].

In recent years, there has been increased interest in the possible role of serotonin in SUDEP (Sudden Unexpected Death in Epilepsy where an attack causes respiratory dysfunction such as apnea). Serotonergic neurons are known to regulate respiration, induce wakefulness, and raise the seizure threshold [46,47]. Hence, serotonin deficiency may contribute to SUDEP. Thus, a series of studies was performed on DBA/1 and DBA/2 mice, in which loud sounds induced seizures followed by respiratory arrest [48,49], that were prevented by the antidepressant – fluoxetine [50].

On the other hand, it was shown that the rats, which are susceptible to audiogenic seizures, have innate deficiency of GABAergic inhibition, especially in the inferior colliculi of the corpora quadrigemina [35]. This leads to the absence of decrease in intensity of the neuronal responses to strong sound signals. Thus, when a strong audiogenic stimulus is received, a large number of neurons of the mentioned brain structure are generally activated followed by the involvement of the overlying brain structures in the formation of generalized seizures. In the animals, which are tolerant to the convulsiogenic effect of a loud sound, however, there is decrease in intensity of neuronal responses to strong sound signals in the inferior colliculi of the corpora quadrigemina by means of GABAergic inhibition. Therefore, even under the effects of strong audiogenic stimuli, the level of activation of the overlying areas of the auditory system in the brain remains rather moderate. Thus, the effect of fluoxetine may not depend on the GABA receptors and be mediated by the subtypes of a lot of receptors [51].

## 5. Conclusion

Given above, we can suppose that decreased manifestation of seizures in the SS rats after acute administration of fluoxetine may be explained by highly distinctive repertoire and spatial distribution of 5-HT receptors in their brain structures in comparison to the ST rats. Specific pattern and level of 5-HT receptor expression, which is high in the ST rats and low in the SS ones, are probably associated with significant innate difference in the levels of 5-HT in neurons of those types of animals. Thus, the anti-seizure effect of fluoxetine in Wistar rats may be highly different based on the innate properties of serotonergic system activity in the brain structures.

The use of animals with a genetically determined type of the nervous system will allow to ascertain the link between this feature and not only relatively simple characteristics of behavior but also the peculiarities of its organization, as well as to open up the prospect of experimental treatment of seizure disorders using pharmacological agents that affect the metabolism of the brain monoamines.

## References

[1] Kuznezova GD, Spiridonov AM (1998). The Mapping of Spike-Wave Discharges in WAG/Rij Rats (a Genetic Strain of Absence Epilepsy). Zh Vyssh Nerv Deiat Im I P Pavlova.

[2] Doretto MC, Fonseca CG, Lobo RB, Terra VC, Oliveira JA, Garcia-Cairasco N (2003). Quantitative Study of the Response to Genetic Selection of the Wistar Audiogenic Rat Strain (WAR). Behav Genet.

[3] Rudolf G, Bihoreau MT, Godfrey RF, Wilder SP, Cox RD, Lathrop M (2004). Polygenic Control of Idiopathic Generalized Epilepsy Phenotypes in the Genetic Absence Rats from Strasbourg (GAERS). Epilepsia.

[4] Ross KS, Coleman JR (2000). Developmental and Genetic Audiogenic Seizure Models:Behavior and Biological Substrates. Neurosci Biobehav Rev.

[5] Jobe PC, Mishra PK, Ludvig N, Dailey JW, Schwartzkroin PA (1993). Genetic Models of the Epilepsies. Epilepsy:Models, Mechanisms, and Concepts.

[6] Malikova LA, Narkevich VB, Klodt PM, Kudrin VS, Raevskii KS, Fedotova IB, Poletaeva II (2008). Effects of the Novel Anticonvulsant Levetiracetam on the Content of Monoamines and their Main Metabolites in the Brain Structures of Rats of the Krushinskii-Molodkina Strain. Neurochem J.

[7] Poletaeva II, Surina NM, Kostina ZA, Perepelkina OV, Fedotova IB (2017). The Krushinsky-Molodkina Rat Strain:The Study of Audiogenic Epilepsy for 65 Years. Epilepsy Behav.

[8] Ismayilova KhYu, Aghayev TM, Semenova TP (2007). Individual Peculiarities of Behavior (Monoaminergic Mechanisms).

[9] Gromova EA, Semenov TP, Chubakov AR, Bobkova NV (1985). Reciprocity of the Relationship between Serotonergic and Noradrenergic Systems of the Brain and its Role in the Regulation of Behavior at Norm and Pathology.

[10] Boer JA (2000). Social Anxiety Disorder/Social Phobia:Epidemiology, Diagnosis, Neurobiology, and Treatment. Compr Psychiatry.

[11] Wong DT, Perry KW, Bymaster FP (2005). Case History:The Discovery of Fluoxetine Hydrochloride (Prozac). Nat Rev Drug Discov.

[12] Shishkina GT (2007). Neuroadaptive Changes in the Brain Under the Effects of the Serotonin Reuptake Blockers. Russ J Physiol.

[13] Kuznezova GD (1998). Audiogenic Seizures in the Different Genetic Strains of Rats. Pavlov J High Nerv Act.

[14] Sarkisova KY, Fedotova IB, Surina NM, Nikolaev GM, Perepelkina OV, Kostina ZA (2017). Genetic Background Contributes to the Co-Morbidity of Anxiety and Depression with Audiogenic Seizure Propensity and Responses to Fluoxetine Treatment. Epilepsy Behav.

[15] Bosak M, Turaj W, Dudek D, Siwek M, Szczudlik A (2015). Depressogenic Medications and Other Risk Factors for Depression among Polish Patients with Epilepsy. Neuropsychiatr Dis Treat.

[16] Shaw FZ, Chuang SH, Shieh KR, Wang YJ (2009). Depression-and Anxiety-Like Behaviors of a Rat Model with Absence Epileptic Discharges. Neuroscience.

[17] Meerson FZ, Mamalyga LM (1994). The Protective Action of Adaptation to Hypoxia in Audiogenic Epilepsy and its Prolongation by using Pharmacologic Agents. Bull Exp Biol Med.

[18] Poletaeva II (2017). Audiogenic Epilepsy in Rodents. Nature.

[19] Shishkina GT, Dygalo NN, Yudina AM, Kalinina TS, Tolstikova TG, Sorokina IV (2006). The Effects of Fluoxetine and Its Complexes with Glycerrhizic Acid on Behavior in Rats and Brain Monoamine Levels. Neurosci Behav Physiol.

[20] Dygalo NN, Shishkina GT, Kalinina TS, Yudina AM, Ovchinnikova ES (2006). Effect of Repeated Treatment with Fluoxetine on Tryptophan Hydroxylase-2 Gene Expression in the Rat Brainstem. Pharmacol Biochem Behav.

[21] Kovalenko IL, Avgustinovich DF, Tolstikova TG (2007). Effects of Single and Chronic Administration of Fluoxetine on Anxiety-Depressive Male and Female Mice. Ross Physiol J.

[22] Simonov PV (1993). Individual Brain. Structural Foundations of Individual Characteristics of Behavior.

[23] Bazyan AS, Grigoryan GA, Ioffe ME (2011). Regulation of Motor Behavior. Adv Physiol Sci.

[24] Salgado-Pineda P, Delaveau P, Blin O, Nieoullon A (2005). Dopaminergic Contribution to the Regulation of Emotional Perception. Clin Neuropharmacol.

[25] Glickstein SB, Schmauss C (2001). Dopamine Receptor Functions:Lessons from Knockout Mice. Pharmacol Ther.

[26] Hervas I, Vilaro MT, Romero L, Scorza MC, Mengod G, Artigas F (2001). Desensitization of 5-HT_1A_ Autoreceptors by a Low Chronic Fluoxetine dose Effect of the Concurrent Administration of WAY-100635. Neuropsychopharmacology.

[27] Furlan PM, Kallan MJ, Have TT, Lucki I, Katz I (2004). SSRIs do not Cause Affective Blunting in Healthy Elderly Volunteers. Am J Geriatr Psychiatry.

[28] Мurphy DL, Lerner A, Rudnick G, Lesch KP (2004). Serotonin Transporter:Gene, Genetic Disorders, and Pharmacogenetics. Mol Interv.

[29] Harkin A, Shanahan E, Kelly JP, Connor TJ (2003). Methylenendioxyamphetamine Produces Serotonin Nerve Terminal Loss and Diminished Behavioural and Neurochemical Responses to the Antidepressant Fluoxetine. Eur J Neurosci.

[30] Dailey JW, Yan QS, Adams-Curtis LE, Ryu JR, Ko KH, Mishra PK (1996). Neurochemical Correlates of Antiepileptic Drugs in the Genetically Epilepsy-Prone Rat (GEPR). Life Sci.

[31] Sparks DL, Buckholtz NS (1985). Combined Inhibition of Serotonin Uptake and Oxidative Deamination Attenuates Audiogenic Seizures in DBA/2J Mice. Pharmacol Biochem Behav.

[32] Freitas RM, Sousa FC, Viana GS, Fonteles MM (2006). Effect of GABAergic, Glutamatergic, Antipsychotic and Antidepressant Drugs on Pilocarpine-Induced Seizures and Status Epilepticus. Neurosci Lett.

[33] Aygun H (2019). The Effect of Fluoxetine on Penicillin-Induced Epileptiform Activity. Epilepsy Behav.

[34] Ferrero AJ, Cereseto M, Reinés A, Bonavita CD, Sifonios LL, Rubio MC (2005). Chronic Treatment with Fluoxetine Decreases Seizure Threshold in Naive but not in Rats Exposed to the Learned Helplessness Paradigm:Correlation with the Hippocampal Glutamate Release. Prog Neuropsychopharmacol Biol Psychiatry.

[35] Treiman DM (2001). GABAergic Mechanisms in Epilepsy. Epilepsia.

[36] Hovorka J, Herman E, Nemcova I (2000). Treatment of Interictal Depression with Citalopram in Patients with Epilepsy. Epilepsy Behav.

[37] Lebedeva AV (2007). Peculiarities of Depression in Patients with Epilepsy. Med J Russ Fed.

[38] Yan QS, Dailey JW, Steenbergen JL, Jobe PC (1998). Anticonvulsant Effect of Enhancement of Noradrenergic Transmission in the Superior Colliculus in Genetically Epilepsy-Prone Rats (GEPRs):A Microinjection Study. Brain Res.

[39] Dovedova EL, Monakov MY (2000). Metabolism of Neurotransmitters in Cortical and Subcortical Brain Structures in Rats with Different Behavioral Characteristics. Bull Exp Biol Med.

[40] Gorbunova AV (2000). The Autonomic Nervous System and Resistance of the Cardio-Vascular Functions to Emotional Stress. Neurochemistry.

[41] Ungar G (1975). Is There a Chemical Memory Trace?. Isr J Chem.

[42] Kulagin DA, Bolondinski VK (1986). Neurochemical Aspects of the Emotional Reactivity and Motor Activity of Rats in New Circumstances. Front Physiol Sci.

[43] Okuhara DY, Beck SG (1994). 5-HT_1А_ Receptor Linked to Inward-Rectifying Potassium Current in Hippocampal CA3 Pyramidal Cells. J Neurophysiol.

[44] Kanner AM (2003). Depression in Epilepsy:Prevalence, Clinical Semiology, Pathogenic Mechanisms, and Treatment. Biol Psychiatry.

[45] Statnick MA, Maring-Smith ML, Clough RW, Wang C, Dailey JW, Jobe PC (1996). Effect of 5, 7-Dihydroxytryptamine on Audiogenic Seizures in Genetically Epilepsy-Prone Rats. Life Sci.

[46] Richerson GB (2004). Serotonergic Neurons as Carbon Dioxide Sensors that Maintain pH Homeostasis. Nat Rev Neurosci.

[47] Bagdy G, Kecskemeti V, Riba P, Jakus R (2007). Serotonin and Epilepsy. J Neurochem.

[48] Faingold CL, Randall M, Zeng C, Peng S, Long X, Feng HJ (2016). Serotonergic Agents act of 5-HT_3_ Receptors in the Brain to Block Seizure-Induced Respiratory Arrest in the DBA/1 Mouse Model of SUDEP. Epilepsy Behav.

[49] Venit EL, Shepard BD, Seyfried TN (2004). Oxygenation Prevents Sudden Death in Seizure-Prone Mice. Epilepsia.

[50] Tupal S, Faingold CL (2006). Evidence Supporting a Role of Serotonin in Modulation of Sudden Death Induced by Seizures in DBA/2 Mice. Epilepsia.

[51] Pasini A, Tortorella A, Gale K (1996). The Anticonvulsant Action of Fluoxetine in Substantia Nigra is Dependent upon Endogenous Serotonin. Brain Res.

